# Antimicrobial stewardship interventions involving community pharmacy teams: a scoping review

**DOI:** 10.1093/jacamr/dlaf156

**Published:** 2025-09-11

**Authors:** Federico Zerbinato, Scott Cunningham, Antonella Pia Tonna

**Affiliations:** School of Pharmacy, Applied Sciences and Public Health, Robert Gordon University, Garthdee House, Garthdee Rd, Aberdeen AB10 7AQ, UK; School of Pharmacy, Applied Sciences and Public Health, Robert Gordon University, Garthdee House, Garthdee Rd, Aberdeen AB10 7AQ, UK; School of Health Sciences, University of New South Wales, High Street, Kensington, Sydney, NSW 2052, Australia; School of Pharmacy, Applied Sciences and Public Health, Robert Gordon University, Garthdee House, Garthdee Rd, Aberdeen AB10 7AQ, UK

## Abstract

**Background:**

The importance of involvement of community pharmacy (CP) teams in antimicrobial stewardship (AMS)-related interventions is justified by the high prevalence of antimicrobial prescribing in primary care. Yet, CP teams are rarely considered as part of AMS activities.

**Aim:**

To synthesize the available evidence in relation to the current involvement of community pharmacists in AMS-related interventions involving CP team members.

**Methods:**

To ensure rigour, the search followed the recommendations of the PRISMA-ScR and the protocol registered with the Open Science Framework. The search was conducted in MEDLINE, International Pharmaceutical Abstracts and CINAHL, identifying studies published between 1999 and 2023 and in English. Studies reporting AMS-related interventions, including at least one CP team member and conducted in a CP setting were included. Study selection and data extraction were performed by two independent reviewers.

**Results:**

Thirty-eight reports were included with pharmacists mainly using patient interviews (*n* = 26) and point-of-care testing (*n* = 15) as information sources to support patient assessment. Pharmacist interventions included providing patient counselling (*n* = 30) and referring to other healthcare professionals (*n* = 17). The main barrier for intervention implementation was the lack of or inadequate remuneration (*n* = 10); the easy accessibility of CPs was the predominant facilitator (*n* = 12). Only three of the included reports were underpinned by implementation theory.

**Conclusions:**

The review is significant since it highlights CP interventions in an area where there is not much evidence. It emphasizes the need to remunerate CPs for their involvement in AMS while highlighting the potential for expansion of easily accessible CP services.

## Introduction

Optimizing the use of antimicrobials is essential, from an individual patient and wider community perspective. As with other medications, ensuring an appropriate use minimizes the risks of adverse drug reactions and drug–drug interactions on an individual patient level; moreover, optimizing the use of antimicrobials has a positive impact on a public health global issue namely antimicrobial resistance (AMR).^1^ This unique feature of affecting both the individual and the community makes antimicrobials particularly suitable for the term ‘antimicrobial stewardship’ (AMS), defined by the WHO as ‘a coherent set of actions which promotes the responsible use of antimicrobials’.^2^

The global emergence and imminent health threat of AMR has highlighted the necessity to urgently optimize the use of antimicrobials. Several countries have initiated health promotion campaigns and have set objectives to tackle AMR^3^; during the 2015 World Health Assembly, countries committed to develop and implement national interventions to tackle AMR through a global action plan.^4^ However, the set goals have not been accomplished yet in high- and middle-income countries and have not been addressed at all in low-income countries.

The most influential global organizations have repeatedly emphasized the importance of targeting AMR; indeed, a United Nations high-level meeting on AMR was held in New York in September 2024,^5^ and the Council of the European Union has recently released a recommendation about the importance of implementing and strengthening AMS programmes.^6^ The document reiterates the necessity of facing and tackling such a challenge in both the community and hospitals, and of including pharmacists in evidence-based measures to support the appropriate use of antimicrobials. The importance of including pharmacists from different practice settings is validated in the most recent statements of the International Pharmaceutical Federation (FIP), in which the necessity of mitigating AMR through AMS involving hospital and community pharmacists is emphasized.^7^

Although almost 80% of antimicrobials consumed are prescribed and dispensed in primary care,^8^ community pharmacy (CP) team members are rarely considered as part of AMS activities. Given the access most patients have to a CP, the involvement of CP team members as part of a structured intervention may support the achievement of the AMS objectives set at the local and international level. Contributing to the evidence on the potential role of CP team members would further inform in an area where the available evidence is limited; community pharmacists have already been identified as an underused resource in AMS with a potential key role in educating the public on the optimal use of antimicrobials.^9,10^

Given the scarcity of knowledge and available literature regarding the involvement of CPs in AMS-related interventions, a scoping review is justified and is the most comprehensive review type to explore and describe the existing interventions and identify potential gaps in the literature.

Other reviews focusing on AMS and CPs have been identified in the literature with interest in knowledge, perceptions and attitudes of community pharmacists towards AMS activities, but with no description provided of AMS-related interventions.^11–13^ Bishop *et al.* provided a narrative overview describing CP interventions to improve antibiotic misuse and overuse, with the review including other outpatient pharmacy settings as it more generally aimed to discuss potential implications for pharmacy training.^14^ In their systematic review, Lambert *et al.* aimed to assess the effectiveness of community pharmacist-led interventions to optimize the use of antibiotics; the review only included randomized controlled trials (RCTs), with the authors recognizing that limiting the inclusion criteria to this study design may not be ideal in the CP setting.^15^

This scoping review adds to the existing literature, as it aims to provide a more comprehensive scenario of the AMS-related interventions specifically conducted in the CP setting to date. Compared with previous similar studies, the review includes interventions involving CP team members, rather than just community pharmacists, recognizing the differences in CP regulation across countries; moreover, the study generally focuses on AMS, with no specific restrictions on antimicrobials.

## Aim and objectives

The aim of this scoping review is to synthesize the available evidence in relation to the current involvement of community pharmacists in AMS-related interventions involving CP team members. Specifically, the outcomes of the review are: (i) to summarize the general characteristics of interventions related to AMS involving CP teams; (ii) to synthesize the evidence relating to structures, processes and outcomes of interventions related to AMS involving CP teams; (iii) to benchmark the identified interventions with the phases and core elements of the UK MRC guidance on developing and evaluating complex interventions and to determine the nature and extent of the use of theory and (iv) to synthesize barriers and facilitators for the implementation of interventions related to AMS involving CP teams.

## Methods

### Protocol and registration

This scoping review follows the recommendations of the Preferred Reporting Items for Systematic Reviews and Meta-Analyses Statement for Scoping Reviews (PRISMA-ScR).^16^ The review protocol was registered with the Open Science Framework.^17^

### Eligibility criteria

For inclusion in the review, studies needed to focus on interventions related to AMS, involving at least one CP team member, independent of the role played (‘led by’ or ‘part of’). Any interventions conducted in a setting different to the CP, including those in an outpatient setting, were excluded. To ensure consistency, the WHO definition of AMS provided was used for inclusion or exclusion purposes.^2^ No geographical restrictions were applied. Studies where antimicrobials were dispensed through sources other than CPs were excluded. Date restrictions were applied with studies published in 1999 and after being included; this reflects the broader scope of practice of the CPs in the last few decades. Since including only RCTs does not represent the optimal study design for a review in this field,^15^ cross-sectional, prospective and retrospective studies, papers reporting empirical data from primary research and qualitative studies were also included. Grey literature, conference abstracts, protocols, book reviews, opinion articles, editorials, letters and reviews, including systematic reviews, were excluded. Papers were considered for inclusion only if they in were in the English language.

### Information sources

To identify the studies relevant to the review and be as comprehensive as possible, the search has involved three different scientific databases with a particular interest in pharmacy and medical sciences: MEDLINE, CINAHL Complete and International Pharmaceutical Abstracts (IPA). The final search was run on 10 December 2023.

### Search

Subject headings were used to run an exhaustive search. Specifically, Medical Subject Headings (MeSH) were identified in MEDLINE and CINAHL headings in CINAHL Complete. Relevant subject headings and keywords were identified through dummy searches conducted to refine the final search strategy. The complete search strategy is described in Supplementary Information (Table S1, available as Supplementary data at *JAC-AMR* Online).

### Selection of sources of evidence

For standardization, consistency and reproducibility purposes the software platform for reviews Covidence was used for data management.^18^ After duplicates were removed, all remaining titles and abstracts were screened by two reviewers independently (F.Z. and A.T. or S.C.), with discrepancies discussed with the third reviewer. Reasons for exclusion were recorded for all abstracts excluded. Full texts were obtained for all included abstracts where possible. Using the same approach, each selected paper was then read by two reviewers (F.Z. and A.T. or S.C.), with discrepancies discussed with a third reviewer. Reasons for exclusion were recorded. A successive manual search was conducted among the relevant excluded references.

### Data charting process and items

A customized data extraction form was created in Covidence by using the Data Extraction 2 tool. Data were extracted independently by two reviewers, with a third reviewer involved when consensus on discordant data was needed. General information about year of publication, country, aims, methods, design and setting (rural, urban or mixed when specified) of each study was collected. The participants, interventions and duration of interventions were identified, together with whether the study was underpinned by a theory. The main findings of all the papers were summarized. To extract relevant and standardized data from each study relating to structures, processes and outcomes of the interventions reported, the Donabedian’s approach for ‘evaluating the quality of medical care’ was used as a reference model^19^: this approach is known for its significant contribution in improving the quality of medical care by considering structures, processes and outcomes of health services, rather than only taking findings into account.^20^ To classify the interventions based on the stage at which they are, the selected studies were benchmarked against the UK MRC guidance for developing and evaluating complex interventions.^21^ The four identified phases were: development, implementation, feasibility and evaluation. Barriers and facilitators for the implementation of the intervention were extracted and mapped to the Consolidated Framework for Implementation Research (CFIR), as described next. The customized data extraction tool was piloted with the first three included reports, sorted alphabetically by first author, and is provided in the Supplementary Information (Table S2).

### Critical appraisal of individual sources of evidence

No critical appraisal of the included reports was conducted, as this is not required for a scoping review.

### Synthesis of results

The heterogeneity of the included studies, given the described eligibility criteria, led to the necessity of a narrative synthesis to summarize the scoping review findings. All the findings were synthesized by a first reviewer following data extraction (F.Z.) and reviewed by a second reviewer (A.T. or S.C.) to ensure consistency in the process. To detail a clear summary, review findings were provided in tables, with the use of validated tools helping to appropriately synthesize the results; the adoption of such tools were discussed and agreed among the three reviewers. The general characteristics of the included papers were first summarized. To further describe the components of the different interventions, the Descriptive Elements of Pharmacist Intervention Characterisation Tool version 2 (DEPICT-2) was adopted; this is a reproducible validated tool, specific to pharmacy and applied in this area of research.^22^ The sections of the tool allowed to summarize the static characteristics of personnel and setting involved in the interventions (structures) and the steps and activities performed in delivering interventions (processes).^23^ Barriers and facilitators to the successful implementation of the identified interventions were mapped against CFIR,^24^ a determinant framework to guide systematic assessment of barriers and facilitators,^25,26^ with use well established in healthcare implementation research.^27^

## Results

### Selection of sources of evidence

**Figure 1.**
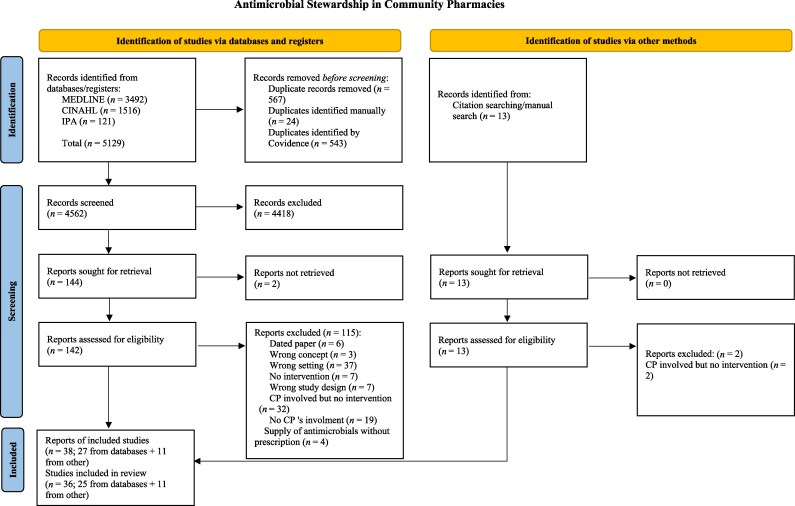
PRISMA chart showing selection steps of the included and excluded reports.

The total number of records identified initially was 5129. The terms records, reports and studies were used in this review as defined by the PRISMA 2020 statement.^28^ Following the removal of duplicates, 4562 records were screened using title and abstract, with 144 reports to be read as full texts. Two records were excluded as their full text was not retrievable. Of the remaining 142 reports, 27 reached the final stage of data extraction. The manual search among the excluded references identified 13 further reports to be read as full texts; two of those were excluded at this stage and 11 reports were included in the review. Three studies had two different reports included, leading to the final inclusion of 38 reports from 36 studies. Figure 1 shows the detailed flow diagram.

### Characteristics of sources of evidence

#### General characteristics of the included interventions

The included reports were published between 2006 and 2023. Most of the studies report interventions conducted in high-income countries, using the 2024–2025 World Bank classification^29^ (12 = USA^30–41^; 9 = UK^42–50^; 3 = Canada^51–53^; 2 = Australia^54,55^; France^56,57^; 1 = Japan^58^; Malta^59^; Poland^60^; Spain^61^ and The Netherlands^62^); one paper was from an upper-middle-income country (Turkey)^63^ and four from lower-middle-income countries (LMICs) (3 = Nigeria^64–66^; 1 = Bolivia^67^). The study designs of the 38 reports identified were 18 non-randomized experimental studies, eight RCTs, seven cohort studies, three cross-sectional studies and two mixed methods studies. All the interventions were delivered by community pharmacists; in six cases other healthcare professionals, including pharmacy assistants (two reports), pharmacy technicians, pharmacy interns and study nurses (one report each), were also involved in the delivery of the intervention. Table S3 in the Supplementary Information describes these and further characteristics of the included interventions.

### Results and synthesis of individual sources of evidence

#### Structures and processes of the interventions

Most of the interventions were based in CPs located in an urban setting. Materials have been classified into those used by patients and those supporting pharmacists to effectively deliver the intervention. In the case of materials for patients, these were more frequently educational leaflets (*n* = 7). Clinical protocols were commonly consulted by pharmacists (*n* = 11). Other resources not specified in the DEPICT-2 were identified as supporting CP staff members in their interventions and were: training (*n* = 21), appropriate private spaces (*n* = 20) and financial incentives (*n* = 9).

Patient interviews (*n* = 26), point-of-care testing (POCT) (*n* = 15) and drug prescription orders (*n* = 15) were the most frequent clinical data sources used and screening results (*n* = 28), drug selection (*n* = 12) and medication adherence (*n* = 11) were the most common variables assessed. Counselling patients was the most common form of intervention delivery (*n* = 30), with referral to another healthcare provider (HCP) or service (*n* = 17) and provision of drug information (*n* = 10) being the other frequently taken actions by the pharmacists. Pharmacists had the autonomy to start antimicrobials or change prescribed antimicrobials in 13 reports.

The focus of the interventions was a specific medical condition in 27 occasions, with 11 focusing on respiratory tract infections (RTIs), seven on HIV, four on urinary tract infections (UTIs), two on hepatitis C virus (HCV), two on tuberculosis and one on malaria. Interventions from the UK included RTIs, UTIs and HCV, while most of the interventions from the USA focused on HIV; the interventions conducted in LMICs were focused on malaria, HIV and tuberculosis. In 10 reports, the intervention focused on a specific pharmacological class (antimicrobials). A pre-specified cohort of patients represented the focus of three interventions (people at risk of acquiring HIV, elderly patients and population already receiving opioid substitute therapy from CP). Table 1 provides a comprehensive overview all the DEPICT-2 components for the identified interventions.

**Table 1.** Structures and processes of the included interventions mapped to the DEPICT-2

Authors, year, country	Recipient	Contact with recipient	Setting (All CPs); urban/rural/mixed	Focus of intervention	Clinical data sources	Variables assessed	Action(s) taken by the pharmacist	Timing of pharmacy contact and frequency	Materials that supported the action	Methods of communication	Changes in therapy and lab tests
Ashiru-Oredope *et al.* 2020, UK^42^	Patient	One-to-one	Mixed	On a specific medical condition: RTIs	Patient interview	Screening results	Patient counselling; referral to other HCP	Once, at an acute patient event	Leaflet to patients; other (training; financial incentive)	Face-to-face; written information where appropriate	
Avong *et al.* 2018, Nigeria^64^	Patient	One-to-one	Urban	On a specific medical condition: HIV (stable patients with successful suppression of viral load on first line ART regimens)	Medical records; drug prescription orders	Medication adherence; drug selection	Patient counselling; other (monitoring of adverse drug reactions)	Medication dispensing, almost every 2 months in a 12 months-period	Other (training; financial incentive)	Face-to-face	
Beahm *et al.* 2018, Canada^52^	Patient	One-to-one	Urban	On a specific medical condition: UTIs	Patient interview; drug prescription orders	Screening results	Patient counselling; change in therapy; referral to other HCP; other (antimicrobial prescribing)	At an acute patient event, follow-up and reassessment at 2 weeks	Guidelines	Face-to-face	Autonomy to start or change prescription medication
Beahm *et al.* 2021, Canada^53^	Patient	One-to-one	Urban	On a specific medical condition: UTIs	Patient interview; drug prescription orders	Screening results	Patient counselling; change in therapy; referral to other HCP; other (antimicrobial prescribing)	At an acute patient event, follow-up and reassessment at 2 weeks	Guidelines	Face-to-face	Autonomy to start or change prescription medication
Beaucage *et al.* 2006, Canada^51^	Patient	One-to-one	Urban	On a specific pharmacological class (oral AB prescription, with treatment lasting between 5–14 days)	Drug prescription orders; patient interview	Drug selection; screening results	Patient counselling; drug information	At an acute patient event, telephone call at day 3, final assessment on last day of antibiotic treatment	Other (financial incentive)	Telephone	
Booth *et al.* 2013, UK^43^	Patient	One-to-one	Mixed	On a specific medical condition: UTIs	Patient interview; drug prescription orders	Screening results	Patient counselling; other (antimicrobial prescribing)	Once, at an acute patient event	Clinical protocol (PGD)	Face-to-face	Autonomy to start prescription medication (PGD)
Buchanan *et al.* 2020, UK^44^	Patient	One-to-one	Not specified	On a specific medical condition: HCV (patients at risk)	Patient interview; POCT	Screening results (also including dried blood spot testing for HCV)	Patient counselling; referral to other HCP	At any time	Other (training; public health campaign)	Face-to-face	
Byrd *et al.* 2019, USA^30^	Patient	One-to-one	Urban	On a specific medical condition: HIV care (documented HIV diagnosis)	Medication list; pharmacy records; laboratory tests; patient interview	Medication adherence; screening results; medication safety	Patient counselling; drug information; monitoring results report; referral to other HCP	Scheduled appointments, with quarterly follow-up visits	Medication action plan; other (training; appropriate private spaces)	Face-to-face	
Demorè *et al.* 2018, France^56^	Patient	One-to-one	Mixed	On a specific medical condition: RTIs (GAS infections)	Patient interview; POCT	Screening results (also including Centor score and RATs)	Patient counselling; referral to other HCP	Once, at an acute patient event	Leaflet to patients; other (training; financial incentive)	Face-to-face	
Göktay *et al.* 2013, Turkey^63^	Patient	One-to-one	Urban	On a specific pharmacological class (oral AB prescription, patients aged ≥18)	Drug prescription orders	Medication adherence; drug selection	Drug information	Medication dispensing	Written reminders to patients; other (warning stickers to patients)	Face-to-face	
Havens *et al.* 2019, USA^31^	Patient	One-to-one	Urban	On a pre-specified sociodemographic patient's characteristic (people at risk of acquiring HIV)	Patient interview; POCT	Screening results (also including baseline parameters, HIV, STIs); medication adherence	Patient counselling; other (antimicrobial prescribing)	At any time, follow-up every 3 months through walk-in	Clinical protocols (CPA); other (training; appropriate private spaces)	Face-to-face	Autonomy to start prescription medication (CPA)
Hayes *et al.* 2022, UK^45^	Patient	One-to-one	Mixed	On a specific pharmacological class (any ABs)	Drug prescription orders; patient interview	Patient educational needs; drug selection; screening results	Patient counselling	Once, at an acute patient event	Guidelines (local AB checklist); leaflet to patients; other (training)	Face-to-face	
Heringa *et al.* 2017, The Netherlands^62^	Patient; GP	One-to-one	Mixed	On a pre-specified sociodemographic patient's characteristic (elderly patients). On a specific pharmacological class (oral ABs)	Drug prescription orders; alert system; POCT	Drug selection; medication safety; screening results (testing for creatinine)	Suggestion of change in therapy (to be agreed with prescriber)	Medication dispensing	Clinical protocol to GPs; guidelines; leaflet to patients; other (training)	Face-to-face	
Hess *et al.* 2009, USA^32^	Patient	One-to-one	College campus CP	On a specific medical condition: tuberculosis	Pharmacy records	Medication adherence; medication safety	Patient counselling	Scheduled appointments	None	Face-to-face	
Hirsch *et al.* 2009, USA^34^	Patient	One-to-one	Not specified	On a specific medical condition: HIV care (patients with at least 1 prescription for ART and an HIV/AIDS diagnosis)	Medication list; pharmacy records; patient interview	Medication adherence; medication safety; therapy effectiveness; screening results	Patient counselling; drug information	Medication dispensing	Other (financial incentive; appropriate private spaces)	Face-to-face	
Hirsch *et al.* 2011, USA^33^	Patient	One-to-one	Not specified	On a specific medical condition: HIV care (patients with at least 1 prescription for ART and an HIV/AIDS diagnosis)	Medication list; pharmacy records; patient interview	Medication adherence; medication safety; therapy effectiveness; screening results	Patient counselling; drug information; monitoring results report	Medication dispensing	Other (financial incentive; appropriate private spaces)	Face-to-face; telephone	
Ikwuobe *et al.* 2013, Nigeria^65^	Patient	One-to-one	Not specified	On a specific medical condition: malaria	Patient interview; POCT	Screening results (also including RATs)	Patient counselling; other (antimicrobial supply)	Once, at an acute patient event	Other (training; appropriate private spaces)	Face-to-face	Autonomy to start prescription medication
Kawachi *et al.* 2017, Japan^58^	Patient	One-to-one	Urban	On a specific medical condition: RTIs (Influenza)	Patient interview; POCT	Screening results (also including RATs)	Patient counselling; referral to other HCP	Once, at an acute patient event	Written action plan; other (training)	Face-to-face; mail-in surveys follow-up on Day 14	
Klepser *et al.* 2016, USA^35^	Patient	One-to-one	Not specified	On a specific medical condition: RTIs (GAS infections)	Patient interview; POCT	Screening results (also including Centor/McIsaac scores, RATs, baseline parameters)	Patient counselling; referral to other HCP; other (antimicrobial prescribing)	At an acute patient event, follow-up by telephone after 24–48 hours	Clinical protocol (CPA); other (financial incentive; training; appropriate private spaces)	Face-to-face; telephone	Autonomy to start prescription medication (CPA)
Klepser *et al.* 2019, USA^36^	Patient	One-to-one	Not specified	On a specific medical condition: RTIs (GAS infections and Influenza)	Patient interview; POCT	Screening results (also including Centor/McIsaac scores, RATs)	Patient counselling; referral to other HCP; other (antimicrobial prescribing)	At an acute patient event, follow-up by telephone after 24–48 hours	Clinical protocol (CPA); other (financial incentive; training; appropriate private spaces)	Face-to-face; telephone	Autonomy to start prescription medication (CPA)
Lambert *et al.* 2005, Bolivia^67^	Patient	One-to-one	Urban	On a specific medical condition: tuberculosis	Patient interview	Screening results	Referral to other service	Once, at an acute patient event	Referral letter (standard referral slip)	Face-to-face	
Madaras-Kelly *et al.* 2006, USA^37^	Patient; Primary Care Provider	One-to-one	Rural	On a specific pharmacological class: broad spectrum antimicrobials. On a specific medical condition: RTIs	Drug prescription orders; patient interview; aggregated clinical decision support system	Drug selection; screening results	Suggestion of change in therapy	Once, at an acute patient event	Clinical protocol (through clinical decision support system); other (appropriate private spaces)	Face-to-face (with patient) telephone (with prescriber)	
Mantzourani *et al.* 2022, UK^46^	Patient	One-to-one	Mixed	On a specific medical condition: RTIs (GAS infections)	Patient interview; POCT	Screening results (also including Centor/FeverPAIN scores)	Patient counselling; referral to other HCP; other (antimicrobial prescribing)	Once, at an acute patient event	Clinical protocol (care pathway); appropriate private spaces	Face-to-face	Autonomy to start prescription medication
Merks *et al.* 2019, Poland^60^	Patient	One-to-one	Not specified	On a specific medication (Amoxicillin/Amoxicillin and Clavulanic Acid used twice daily)	Drug prescription orders	Drug selection; medication adherence	Drug information	At an acute patient event, final interview at the end of AB course	Pictorial information to patients	Face-to-face; telephone	
Munoz *et al.* 2014, Spain^61^	Patient	One-to-one	Urban	On a specific pharmacological class (oral AB prescription, patients aged ≥18)	Drug prescription orders; patient interview	Drug selection; patient educational beliefs; medication adherence; screening results	Drug information	At an acute patient event, final interview one week after dispensation	Guidelines (dispensing protocol); other (appropriate private spaces)	Face-to-face; telephone	
Murphy *et al.* 2012, USA^38^	Patient	One-to-one	Not specified	On a specific medical condition: HIV care (treatment naïve and established patients)	Pharmacy Records	Medication adherence	Patient counselling	Medication dispensing	Other (training; appropriate private spaces)	Face-to-face	
Northey *et al.* 2015, Australia^54^	Patient	One-to-one	Not specified	On a specific pharmacological class (AB prescription)	Drug prescription orders	Drug selection	Drug information	At an acute patient event, final interview one month after dispensation	Leaflet (Australian National Prescribing Service) to patients	Face-to-face; telephone	
O'Neill *et al.* 2022, UK^47^	Patient	One-to-one	Not specified	On a specific medical condition: RTIs	Patient interview; POCT	Screening results (also including CRP testing)	Patient counselling; referral to other HCP	Once, at an acute patient event	Guidelines (service outline); other (training; appropriate private spaces)	Face-to-face	
Onwunduba *et al.*, 2023, Nigeria^66^	Patient	One-to-one	Urban	On a specific medical condition: RTIs	POCT	Screening results (CRP testing)	Patient counselling; referral to other service	Once, at an acute patient event	Other (training; appropriate private spaces)	Face-to-face	Autonomy to start prescription medication
Pham *et al.* 2013, USA^39^	Patient	One-to-one	Urban	On a specific pharmacological class (AB prescription of one of 18 commonly prescribed ABs)	Drug prescription orders	Drug selection	Patient counselling; drug information	At an acute patient event, follow-up by telephone after 5–7 days	Auxiliary labels to patients; other (appropriate private spaces)	Face-to-face; telephone	
Radley *et al.* 2017, UK^48^	Patient	One-to-one	Mixed	On a specific medical condition: HCV. On a pre-specified sociodemographic patient’s characteristic (population already receiving opioid substitute therapy from CP)	Patient interview; POCT	Screening results (also including dried blood spot testing for HCV)	Patient counselling; other (antimicrobial prescribing)	Medication dispensing (opioid substitution therapy)	Clinical protocol (care pathway); other (training; appropriate private spaces)	Face-to-face	Autonomy to start prescription medication (pharmacist independent prescriber)
Shrestha *et al.* 2020, USA^40^	Patient	One-to-one	Urban	On a specific medical condition: HIV care (patients already on or planning to start ART)	Medication list; medical records	Medication adherence; therapy effectiveness; medication safety	Drug information; patient counselling; monitoring results report; update of patient’s medication list	Scheduled appointment, quarterly follow-ups	Other (training; appropriate private spaces)	Face-to-face	
Sim *et al.* 2021, Australia^55^	Patient	One-to-one	Urban	On a specific medical condition: RTIs	Patient interview; POCT	Screening results (also including CRP testing)	Patient counselling; referral to other HCP	Once, at an acute patient event	Other (training; appropriate private spaces)	Face-to-face; telephone	
Thornley *et al.* 2016, UK^49^	Patient	One-to-one	Urban	On a specific medical condition: RTIs (GAS infections)	Patient interview; POCT	Screening results (also including Centor scores and RATs)	Patient counselling; referral to other HCP; other (antimicrobial prescribing)	Once, at an acute patient event	Clinical protocol (PGD); Other (financial incentive; training; appropriate private spaces)	Face-to-face	Autonomy to start prescription medication (PGD)
Thornley *et al.* 2020, UK^50^	Patient	One-to-one	Urban	On a specific medical condition: UTIs	Patient interview; other (urine dipstick self-test)	Screening results (also including self-test results)	Patient counselling; referral to other HCP; other (antimicrobial prescribing)	At an acute patient event, final evaluation after 72 hours	Self-monitoring device to patients; leaflet to patients; clinical protocol (PGD); other (training)	Face-to-face	Autonomy to start prescription medication (PGD)
Treibich *et al.* 2017, France^57^	Patient	One-to-one	Mixed	On a specific pharmacological class (AB prescription of one of 14 commonly prescribed ABs)	Drug prescription orders	Drug selection	Other (provision of exact number of antibiotic tablets)	Medication dispensing, follow-up after 2–3 days	Other (structures to provide per-unit dispensing)	Face-to-face; telephone	
Tung *et al.* 2018, USA^41^	Patient	One-to-one	Urban	On a specific medical condition: HIV care (PrEP service)	Patient interview; pharmacy records; POCT (HIV, serum creatinine, hepatitis serology, pregnancy)	Screening results (also including testing for HIV, creatinine, hepatitis)	Patient counselling; referral to other HCP; other (antimicrobial prescribing)	Scheduled appointment, follow-up at 1 month and 3 months thereafter	Clinical protocol (CDTA); other (appropriate private spaces; training)	Face-to-face; telephone; emails	Autonomy to start prescription medication (CDTA)
West and Cordina, 2019, Malta^59^	Patient	One-to-one	Urban	On a specific pharmacological class (oral AB prescription, patients aged ≥ 18)	Drug prescription orders	Drug selection	Patient counselling	At an acute patient event, follow-up the day after expected treatment completion	Educational leaflet to patients; other (appropriate private spaces)	Face-to-face; telephone	

AB, antibiotic; ART, antiretroviral therapy; CDTA, Collaborative Drug Therapy Agreement; CPA, Collaborative Practice Agreement; CRP, C-reactive protein; GAS, group A streptococcus; PGD, patient group direction; PrEP, pre-exposure prophylaxis; RAT, rapid antigen test; STI, sexually transmitted infections.

#### Outcomes of the interventions

On the basis of the classification suggested by Newham and colleagues in 2023, the outcomes measured in the included studies (Table S3, Supplementary Information) can be divided into four categories: (i) service outcomes, including medication and healthcare optimization and population-level monitoring of health-related targets; (ii) humanistic outcomes, including medication adherence, health-related quality of life, health status, self-efficacy and lifestyle; (iii) clinical outcomes, including disease indicators, achieving monitoring targets, disease presentation and risk and physical characteristics and (iv) economic outcomes, including healthcare costs and cost-effectiveness.^68^ Most of the interventions in the review were service outcomes (30/38); these types of outcome were often related to medication optimization, with CP-based interventions leading to a more appropriate use of antimicrobials according to local guidelines in many cases^35,36,43,45,49,50,53,56,65,66^; other service outcomes were related to healthcare optimization, as demonstrated by several interventions reducing unnecessary appointments with physicians,^42,46,49,50,56^ and monitoring of health-related targets, as in the case of interventions reporting POCT results and positivity ratio.^35,36,47,48,55,58^ Humanistic outcomes were reported in 17 interventions, with medication adherence being addressed in all of them; most of these interventions reported improvements in the groups of patients attending the CP compared with those who did not, except for three interventions reporting no significant differences between groups of patients following the CP or the conventional pathway.^39,51,60^ Clinical (*n* = 8) and economic (*n* = 6) outcomes of interventions were also reported. Clinical outcomes were focused on clinical cure or rates of adverse events, with findings being mainly descriptive and not reporting comparisons between groups; in three cases, CP-based interventions led to reductions in costs compared with conventional pathways.^34,40,48^ Table S3 in the Supplementary Information includes all the identified outcomes of the included studies.

#### Phases of interventions as described by the UK MRC guidance and use of theory

Almost all the included reports described interventions in the phase of evaluation (*n* = 20) or feasibility (*n* = 16), as described by the UK MRC guidance. The use of theoretical approaches was mentioned and described in only three reports. The COM-B model, a behaviour change framework in which capacity, opportunity and motivation are proposed as necessary components for any behaviour to occur,^69^ was used in the implementation of two interventions. The Normalisation Process Theory, a framework identifying factors that facilitate and inhibit the incorporation of an intervention into daily practice,^70^ was adopted in one intervention (Table S3, Supplementary Information).

#### Barriers and facilitators to implementation of the interventions

When identified in the reports, barriers and facilitators for the implementation of the interventions were mapped against the CFIR.^26^ The first domain (Innovation) focused on the aspects of AMS-related interventions being implemented: most of the barriers within this domain regarded complexity of patient care in specific patient cohorts (*n* = 4) and costs of implementing intervention including required technologies (*n* = 3). However, having interventions that were considered simple, inexpensive or cost-effective (*n* = 5) was deemed easier to implement.

Several barriers and facilitators were identified within the second domain (Outer setting), which in this review refers to external connections between CPs and relevant stakeholders. Financial issues represented a dominant construct, with the lack of adequate remuneration being reported as a barrier for intervention implementation in 10 cases. ‘Local attitudes’ were often considered as enablers for intervention implementation, all associated with patient-related factors, such as patient engagement and trust due to pre-existing relationships (*n* = 7). Most of the facilitators fell within the ‘Local conditions’ construct, with easy CP accessibility being reported 12 times.

In the third domain (Inner setting), which in this review refers to the CP, ‘available resources’ and ‘access to knowledge and information’ were often reported as barriers; lack of time significantly impacting CP workload (*n* = 7) was the most common barrier in terms of available resources and lack of training in intervention delivery (*n* = 3) resulted in lack of access to knowledge and information.

Barriers and facilitators belonging to the fourth domain (Individuals) were mostly related to the community pharmacists as deliverers of the interventions. A lack of confidence in delivering the intervention or querying prescribing decisions was reported as a barrier (*n* = 3), but pharmacists showed an intrinsic motivation to deliver the intervention and take on expanded roles (*n* = 4) potentially facilitating the implementation of AMS-related interventions.

Table 2 summarizes all reported barriers and facilitators.

**Table 2.** Barriers and facilitators for the implementation of the included interventions mapped against the CFIR

Barriers (references)	CFIR domains and constructs*	Facilitators (references)
I—Innovation		
B. Lack of evidence supporting the intervention outcomes^32^	Innovation evidence-based	B. Contributing to development of evidence-based practice^35,52,53^
C. None	Innovation relative advantage	C. High quality diagnostic tests^36^; reduced specialist clinic attendance requirements^48^
D. Lack of generalisability to other cohorts^32^	Innovation adaptability	D. Applicability to similar settings^32^; model scalability^32,34^; flexibility across CPs^55^
F. Complexity of patient care in specific patient cohorts^33,34,48,67^	Innovation complexity	F. None
G. None	Innovation design	G. An active intervention as opposed to a passive intervention^53^; comprehensive well-designed intervention^33–35^; potential for clinical services becoming a new revenue stream for CPs^41^
H. Costs of implementing intervention including required technologies^34,36,66^	Innovation cost	H. Simple, inexpensive and cost-effective intervention that is affordable^33,35,48,51,55^
II—Outer setting		
B. Low acceptance of pharmacist implemented intervention by some patients with potential mistrust^48,52,67^; pharmacists’ concern of harming relationships with physicians^37,53^; low level of health literacy in recipients^39^	Local attitudes	B. Patient engagement and trust particularly with vulnerable groups due to pre-existing relationships^30,38,44,46,48,52,56^; patients’ willingness to pay^31,55^; patient recall of advice^38^; patient adherence to pharmacist recommendation^32^; patient awareness^46,56^
C. Lack of accessibility of CP in rural settings^64^	Local conditions	C. Easy CP accessibility^31,35,41,44,46,48–50,52,64–66^; potential savings for healthcare systems in the long term^34,49,57^; intervention through an already existing system/healthcare worker^48,65^; positive impact on the environment due to potential decrease of wastage-associated costs and leftover antibiotics^57,59^
D. Lack of well-established interprofessional collaboration with GPs^52^; lack of access to clinical data or information to implement intervention^48,51,62^; lack of continuity and communication across primary care pathway^45^; lack of access to patient notes^45^	Partnerships and connections	D. Collaboration with pharmacists’ association^67^; healthcare system support to CPs^67^; interprofessional collaboration with primary care providers^38,40,55^
E. Limited ability for pharmacists to prescribe independently or make independent decisions^35,52^	Policies and laws	E. None
F. Lack of adequate remuneration for delivery of intervention^30,31,33,40,41,44,47,49,50,56^; lack of insurance coverage or consultation fee requiring patients to pay for intervention^31,67^	Financing	F. Financial incentive to deliver intervention^37^; potential fundings^55^
G. •1. Lack of awareness and promotion of services^47,55^ •2. Against interest of pharmaceutical companies^57^	External pressure: Societal Market	G. None
III—Inner setting		
A. Logistical challenges to appropriate intervention implementation^31,48^ •1. Inappropriate physical infrastructure leading to lack of confidentiality^41,44^ •2. None	Structural characteristics: Physical infrastructure Information technology infrastructure	A. 1. None 2. IT infrastructure fit to support intervention implementation^62^
F. None	Compatibility	F. Well-integrated intervention in CP routine and workflow^37,45,55^
J. Lack of time significantly impacting CP workload^36,40,42,44,47,57,65^; limited CP capacity for large scale roll-out^64^; resource-intensive^34^ •1. Lack of funding for appropriate materials and equipment^36^ •3. Lack of educational materials^37^	Available resources: Funding Materials and equipment	J. Simple, inexpensive and efficient intervention^36,45,51,56,59^ sufficient clinical capacity^44^ 1. None 3. Educational leaflet to support self-care advice provision and AMR explanation^42^
K. Lack of training in intervention delivery^40,52,55^; local prescribing guidelines required for intervention delivery^45^; risk of missed diagnoses^35^	Access to knowledge and information	K. Clear protocols supporting intervention^36^
IV—Individuals: roles subdomain (characteristics subdomain)		
B. (C. Opportunity) Acceptance of intervention by CP leadership^31^	Mid-level leaders	B. None
E. None	Implementation leads	E. (D. Motivation) Intrinsic motivation to deliver intervention^48^
F. None	Implementation team members	F. (B. Capability) Increased staff knowledge^45^ (D. Motivation) Increased staff motivation^45^
H. (B. Capability) Lack of confidence to deliver intervention or in querying prescribing decisions^31,45,52^ (C. Opportunity) Limited clinical role due to a need to focus on medication supply^60^	Innovation deliverers	H. (B. Capability) Appropriate training to deliver intervention^42,44,46^; experience on blood testing^66^ (D. Motivation) Intrinsic motivation to deliver intervention and take on expanded roles^43,48,59,67^
V—Process		
B. •1. Lack of profit from antimicrobials’ sales if these are not dispensed as part of intervention^65,67^	Assessing needs: Innovation deliverers	B. None
C. Difficulty in explaining AMR in lay terms to patients^42^; language barriers^32,45^; antimicrobial collected by representatives making the intervention not possible^32^	Assessing context	C. None
D. Concerns around sustainability^30,64^	Planning	D. Whole team involvement^45^
F. •1. Variation in CP uptake^48^	Engaging: Innovation deliverers	F. None
H. None	Reflecting and Evaluating:	H. Patient satisfaction with intervention delivery^47,55^

*CFIR domains:
Innovation = aspects of the AMS-related interventions being implemented Outer setting = external connections between CPs and relevant stakeholders Inner setting = CP Individuals = roles and characteristics of individuals, with community pharmacists being innovation deliverers and patients being innovation recipients Process = activities and strategies used to implement AMS-related interventions

*Constructs with no identified barriers or facilitators have not been included in the table.

## Discussion

### Summary of evidence

#### Key findings

This scoping review identified 38 reports, from 36 different studies, describing AMS interventions including at least one CP team member. Delivery of interventions was facilitated by the availability of appropriate private spaces in CPs. Training was required and provided in most cases, with pharmacists using clinical protocols or guidelines to inform decision making and providing educational leaflets to patients. To inform patient assessment, patient interview, POCT and drug prescription orders were the main sources of information, with subsequent screening of results mainly supporting pharmacist decision making as part of the intervention delivery. Service outcomes were mainly reported, with less focusing on clinical and economic outcomes. The interventions were generally at the stage of feasibility or evaluation, as described the UK MRC guidance.^21^ Only three interventions reported the use of theoretical frameworks or models to underpin interventions. Dominant barriers for the implementation of the interventions were related to the lack of an adequate remuneration for CP services; the ease of access of CPs was identified as the most common facilitator.

#### Interpretation of findings

The review contributes to the already growing evidence of the expanding role of the community pharmacist beyond the supply of medications; this finding is consistent with literature from other therapeutic areas, such as cardiovascular disease, diabetes and asthma,^71–73^ where community pharmacists contribute to the wider patient care. The focus of the included interventions varied from country to country: most interventions reported were from the USA with a particular interest in HIV. The finding may reflect the increasing prevalence of the infection in the USA over the last few years, which is higher compared with European countries.^74^ It is also likely to reflect the increased attempt to address social and cultural inequities in the USA, where new HIV diagnoses are higher in black and socially vulnerable populations.^75^ As described by a recent review conducted in the USA, efforts are required to create effective clinical and social services to meet the needs of vulnerable populations, such as HIV patients^76^; in this regard, the easy access of CPs and pre-existing relationships with vulnerable patients, highlighted as facilitators to intervention implementation in this review, makes CPs an attractive healthcare resource in the field. Further research may clarify whether the involvement of CPs in HIV-targeted activities could be beneficial in Europe, too.

The other dominant country emerging in this review is the UK. This finding is in line with the efforts being made by the National Health Service (NHS) in the UK to increase AMS-related activities in CPs, as most of the antimicrobials are prescribed in primary care.^77^ Indeed, the NHS Pharmacy First Service in England and Wales and the NHS Pharmacy First Plus Service in Scotland were launched recently, enabling CPs to be involved in the management of common infections and supply the most appropriate medication.^78,79^

Most interventions focused on RTIs, an ideal target for AMS in CPs, considering the high prevalence and frequency of inappropriate prescriptions for antibiotics for self-limiting viral infections in primary care.^80^ Often, where the focus was RTIs, patients were initially screened using validated scores (such as the Centor score) followed by performance of rapid tests, with positive service outcomes. These findings confirm the growing international interest in delivery of POCT through CPs.^81^ Reducing unnecessary antimicrobial prescribing through existing healthcare workers and supported by validated tools is consistent with the need to effectively fight AMR worldwide and has been highlighted by numerous international organizations.^6,7^

Interventions focusing on UTIs were based in countries where pharmacists can prescribe antibiotics informed by local guidelines, such as the UK and Canada. In these reports, pharmacists were more likely to adhere to guidelines in prescribing compared with physicians.^43,53^ The finding encourages a wider involvement of CPs in the management of UTIs, which appears both feasible and appropriately addressed by community pharmacists and reinforces the growing interest in pharmacist prescribing as an opportunity to optimize antimicrobial therapies.

An overall low number of reports have been identified from LMICs; yet, AMS-related interventions in CPs may represent a relevant goal in these countries too, where the ease of CP access was reported as a facilitator for implementation.^64–66^ This is even more significant taking into account the difficult access to primary care physicians in such countries.^82^ AMS-related interventions may also be of particular significance given that the supply of antibiotics without prescription is a common practice in LMICs.

Clinical outcomes were not widely reported. A recent umbrella review looking at the impact of community pharmacist-led interventions in the management of chronic diseases showed significant improvements in a variety of clinical outcomes.^83^ The finding of our review may suggest that service outcomes, such as antimicrobial prescribing rates, may be prioritized over clinical outcomes in AMS-related interventions. Focusing on clinical outcomes as a consequence of AMS-related interventions in CPs may increase their relevance and is recommended in future research. Moreover, economic outcomes were often not considered in the included studies, with interventions providing indication on costs mainly focusing on HIV and HCV.^33,34,40,44,48^ The finding may reflect the structured involvement of CPs in comprehensive interventions addressing these diseases and the financial burden carried by these two infections leading to a high need of cost-effective interventions^84,85^; as for HCV, the two reports come from the UK where CPs are considered essential in the elimination of hepatitis C by 2030, as targeted by the WHO at an international level.^86^ The intervention costs and lack of remuneration for CPs have been often reported as barriers for the intervention implementation; providing positive economic outcomes in additional AMS-related interventions in CPs, such as those on RTIs, may represent a fundamental challenge for future implementation.

The fact that few reports provided economical outcomes relating to the interventions may also be linked to the paucity of reports describing the development or implementation stages of the interventions, with most included interventions being at the pilot phase. Keeping in mind the four key stages for complex intervention research as outlined by the MRC (development, feasibility and piloting, evaluation and implementation and dissemination),^21^ results may imply that the implementation phase has not yet been achieved, and this is fundamental in embedding the service into routine practice. Following on, the positive service or humanistic outcomes of several interventions at the feasibility or evaluation phase may require to be supported by further studies addressing implementation challenges on a broader scale and associated to the real-world context. The authors of this review have found classification of the intervention stages according to the UK MRC guidance challenging, since the identification of the stage described in the reports did not always match with the interpretation provided in the included reports. Although the definitions of the different phases provided by the MRC are clear, further strategies to align the interpretation of different researchers may be beneficial.

Furthermore, only three reports were underpinned by theory with all being at the evaluation phase. Interestingly, a very recent scoping review summarizing behavioural frameworks, models and theories used in pharmacy practice research reported a growing use of theory, especially in interventions at their development phase as defined by the UK MRC.^87^ This is not consistent with findings of our review, but the studies included in the review by Nazar *et al.* are not focused on AMS and not entirely conducted in the community setting, suggesting a need to also develop AMS-related interventions in CPs underpinned by theory. The use of theory underpinning pharmacy-based research, particularly at the early stages of interventions, enhances robustness and relevance, leading to a more likely successful implementation into practice.^24,88^

Several facilitators for intervention implementation were identified as belonging to the ‘local attitudes’ and ‘local conditions’ within the ‘Outer setting’ of the CFIR. Data from the USA indicate that 88.9% of the population lives within 5 miles of a CP,^89^ which may differ from country to country but is representative for CP proximity to the general population. The findings of our review confirm how CPs are not only well positioned for medication supply, but also well suited for being involved in health-related interventions, such as those focusing on AMS.

Patient engagement due to pre-existing relationships, particularly with vulnerable groups, proved to be a facilitator. However, ‘Partnerships and connections’ with physicians were more challenging.^37,38,40,45,52,53,55^ In a recent study exploring views of general practitioners (GPs) and community pharmacists regarding their potential collaboration in implementing AMS programmes, Saha *et al.* showed that an effective interaction was supported by both the healthcare professionals^8^; however, GPs may also perceive barriers in this regard, such as reluctance in sharing patient care with community pharmacists and concerns over clinical competences of pharmacists.^90^ The findings of our review further highlight the need to improve trust and collaboration between community pharmacists and GPs to implement AMS-related interventions; the reduction in the number of unnecessary visits to GPs demonstrated by some of the interventions described in this review may encourage GPs in exploring the potential of collaborating with community pharmacists in AMS-related activities.^42,46,50^

The lack of an adequate remuneration to deliver the intervention was a common barrier in our review. The finding is consistent with the results of several other studies investigating aspects related to the remuneration of different types of CP service, which all identify the lack of an adequate remuneration as a major barrier affecting service sustainability.^27,91,92^ Nevertheless, community pharmacists proved to be motivated to be involved in AMS^43,48,59,67^; such intrinsic motivation, coupled with patient engagement and the easy CP accessibility, is a facilitator that should support efforts to incorporate AMS-related interventions into remuneration schemes.

The lack of time and the impact of AMS-related interventions on the CP workload were the most frequently reported barriers to intervention implementation. In a review published in 2012, Lea and colleagues addressed the growing stress of community pharmacists due to the increased workload in CPs^93^; the review was based in the UK where the implementation of CP services was in its infancy. Twelve years on this still seems to be a significant barrier, as confirmed by a Delphi study involving community pharmacists across Europe,^94^ where an expansion of practice, including appropriate times dedicated to AMS-related activities, is still a challenge. The finding of our review further reiterates that time constraints are a general barrier for the implementation of AMS-related interventions, as shown by the fact that reports are based in a variety of different countries with different incomes.

Our review highlights a lack of AMS implementation leads in the CP setting, which could have been expected given the paucity of studies focused on AMS in CPs. AMS programmes are essential in hospitals, where pharmacists are also appointed as ‘champions’ to ensure the successful implementation of those programmes.^95^ The identification of AMS leaders in the community setting may represent a fundamental development for advancing implementation in this setting rather than solely in a hospital environment.

### Strengths and limitations

The lack of geographical restrictions in inclusion of reports in this scoping review reflects the global need of AMS-related initiatives, as highlighted by global organizations and described in the introduction. However, this may also represent a limitation, as the legal and regulatory framework for CPs and their scope of practice varies from country to country, with findings that cannot always be applied from study to study; moreover, the focus of some interventions based on infection epidemiology may be relevant for some countries and irrelevant in others.

A strength of this review was the inclusion of several possible study designs, as the scarcity of literature in the field required the search to be as comprehensive as possible. The use of validated tools and frameworks, such as the DEPICT-2 and CFIR, represent a further strength for this scoping review. The specificity of DEPICT-2 in describing pharmacist interventions allowed to describe the interventions effectively and in detail; however, a potentially more specific tool in the CP setting may be helpful and build a basis for much needed structured interventions within CP. The extensive use of the CFIR in research ensured a suitable and systematic approach in detecting barriers and facilitators for the implementation of AMS-related interventions in CPs.

### Conclusions

The integration of community pharmacists in AMS-related interventions is an essential but widely unaccomplished challenge. The findings from this review show a variety of therapeutic areas in which community pharmacists can contribute to AMS with different roles, with generally positive service outcomes leading in most cases to an optimized use of antimicrobials. To support these findings, ensure robustness of interventions and successfully implement into clinical practice, future AMS-related interventions in CPs should be underpinned by theory; consideration of clinical outcomes may also support the importance of involving CP teams in AMS. Furthermore, the review highlights and confirms the need of incorporating CPs in remunerative schemes when involved in AMS; such involvement is facilitated by positive relationships between community pharmacists and patients, but future research may look towards building relationships of trust between community pharmacists and GPs to work collaboratively in AMS-related interventions.

## Supplementary Material

dlaf156_Supplementary_Data

## Supplementary data

Tables S1–S3 are available as Supplementary data at *The Journal of Antimicrobial Chemotherapy* Online.
