# Supplementary material for: Antimicrobial stewardship interventions involving community pharmacy teams: a scoping review
Source: JAC Antimicrob Resist. 2025 Sep 11;7(5):dlaf156. doi: 10.1093/jacamr/dlaf156 (PMC12455195; doi:10.1093/jacamr/dlaf156)
Supplement: dlaf156_Supplementary_Data [file dlaf156_supplementary_data.zip › Supplementary information - Search Strategy - Table S1.docx]

**Table S1**: Search strategy used for identification of studies

| **Core term** | **Sub-terms or MESH terms (MEDLINE/CINAHL)** | **MEDLINE**  **(first or only word in brackets)** | **IPA (only keywords)** | **CINAHL**  **(second or only word in brackets)** | **TOTAL** |
| --- | --- | --- | --- | --- | --- |
| 1. **Community Pharmacy** | Pharmacies (MESH/keyword) OR Community Pharmacy Services (MESH/keyword) OR Primary Health Care (MESH) OR Evidence-Based Pharmacy Practice (MESH/keyword) OR Community Health Services (MESH) OR Pharmacy, Retail (keyword/MESH) OR Pharmacy Service (keyword/MESH) OR “Community Pharm*” (keyword) OR “Chemist Shop” (keyword) | **534 266** | **19 724** | **576 611** | **1 130 601** |
| **2. Community Pharmacy Team** | Pharmacists (MESH) OR Pharmacy Technicians (MESH) OR Patient Care Team (MESH/keyword) OR Multidisciplinary Team (keyword/MESH) OR “Chemist” (keyword) OR “Community dispens*” (keyword) OR “Counter Assistant” (keyword) OR “Pharmacy team*” (keyword) | **109 346** | **59 389** | **73 398** | **242 133** |
| **3. Antimicrobial Stewardship** | Antimicrobial Stewardship (MESH) OR Drug Resistance, Microbial (MESH) OR Anti-Infective Agents (MESH/keyword) OR Anti-Bacterial Agents (MESH/keyword) OR Drug Resistance, Multiple (MESH) OR Antiinfective agents (keyword/MESH) OR “Antimicrobial Safety” (keyword) OR “Antimicrobial Counselling” (keyword) | **918 472** | **1 527** | **195 801** | **1 115 800** |
| **4. Service evaluation** | Health Services Research (MESH) OR  Implementation Science (MESH) OR  Pharmaceutical Services (MESH/keyword) OR  Program Evaluation (MESH) OR  Intervention OR Service Development OR  Service Evaluation OR  Service Implementation OR  Service Process | **1 154 430** | **18 962** | **426 169** | **1 599 561** |
| **(1 OR 2) AND 3** |  | **11 402** | **811** | **12 070** | **24 283** |
| **((1 OR 2) AND 3) AND 4** |  | **3 503** | **110** | **1 516** | **5 129** |
