# Supplementary material for: Antimicrobial stewardship interventions involving community pharmacy teams: a scoping review
Source: JAC Antimicrob Resist. 2025 Sep 11;7(5):dlaf156. doi: 10.1093/jacamr/dlaf156 (PMC12455195; doi:10.1093/jacamr/dlaf156)
Supplement: dlaf156_Supplementary_Data [file dlaf156_supplementary_data.zip › Supplementary information - Data extraction tool - Table S2.docx]

**Table S2**: Data extraction template

| **DATA** | **POSSIBLE OPTIONS** |
| --- | --- |
| **General information** | |
| Study ID |  |
| Title |  |
| Reference |  |
| Lead author contact details |  |
| Date of publication |  |
| Country in which the study is conducted |  |
| Aim of the study |  |
| Study design | - Randomised controlled trial - Non-randomised experimental study - Cohort study - Cross-sectional study - Case-control study - Qualitative research - Prevalence study - Diagnostic test accuracy study - Economic evaluation - Other |
| Methods |  |
| Setting | - Rural - Urban - Mixed - Non specified - Other |
| Participants | - Community pharmacists - Other CP team members - Patients - Other healthcare professionals - Other |
| Intervention |  |
| Duration of intervention |  |
| Use of theory |  |
| Main findings |  |
| **Donabedian approach** | |
| Structures |  |
| Processes |  |
| Outcomes |  |
| **MRC framework** | |
| Stage of the intervention according to the MRC guidance | - Development - Evaluation - Feasibility - Implementation |
| **Barriers and facilitators** | |
| Barriers |  |
| Facilitators |  |
