# Supplementary material for: Antimicrobial stewardship interventions involving community pharmacy teams: a scoping review
Source: JAC Antimicrob Resist. 2025 Sep 11;7(5):dlaf156. doi: 10.1093/jacamr/dlaf156 (PMC12455195; doi:10.1093/jacamr/dlaf156)
Supplement: dlaf156_Supplementary_Data [file dlaf156_supplementary_data.zip › Supplementary Information - Description of interventions - Table S3.docx]

**Table S3**: Description of the general characteristics of the included interventions

| **Authors, year** | **Country** | **Title** | **Aim as stated by the authors** | **Study design** | **Healthcare professional delivering intervention** | **Theory – review objective 3** | **Intervention – stage as for review objective 3 and brief description** | **Outcomes measured/ main findings** |
| --- | --- | --- | --- | --- | --- | --- | --- | --- |
| Ashiru-Oredope et al.,  2020 ^42^ | United Kingdom, England | Improving Management of Respiratory Tract Infections in Community Pharmacies and Promoting Antimicrobial Stewardship: A Cluster Randomised Control Trial with a Self-Report Behavioural Questionnaire and Process Evaluation | To test an intervention to improve the management of RTIs and promote antimicrobial stewardship within a CP. | Cluster Randomised controlled trial | Community pharmacist | Yes (COM-B model) | Evaluation – Educational intervention with pharmacists receiving prior AMS-related training and providing additional patient advice about RTIs supported by a leaflet in the intervention group, and usual care in a control group. | - *Number of pharmacists’ referrals to GPs*: reduced likelihood of GP referrals for infection compared to control group (OR = 0.91; 1726 patients in the intervention group, 1923 in the control group). - *Provision of self-care advice:* greater provision of self-care advice. - *Written information to patients*: patients in the intervention arm were more likely to receive written information if also provided with self-care advice (OR = 3.53; p < 0,01). - *Sign-posting to non-prescription medicines*: not reported. - *Referrals to pharmacists by other CP staff members*: limited evidence of higher number of referrals in the intervention arm (OR = 1.42; p = 0.11). |
| Avong et al., 2018 ^64^ | Nigeria | Integrating community pharmacy into community based anti-retroviral therapy program: A pilot implementation in Abuja, Nigeria. | To assess the feasibility of shifting to using CPs for ART delivery. | Non-randomised experimental study | Community pharmacist | No | Implementation – Patient counselling, ART supply and adherence monitoring in selected CPs. | - *Rate of prescription refill*: excellent (100%). - *Retention in care*: very high (287/290; 99.3%), only one participant was lost to follow-up. |
| Beahm et al.,  2018 ^52^ | Canada | Outcomes of Urinary Tract Infection Management by Pharmacists (RxOUTMAP): A study of pharmacist prescribing and care in patients with uncomplicated urinary tract infections in the community. | To evaluate the effectiveness and safety of, and patient satisfaction with, pharmacist assessment and management of patients with uncomplicated UTI. | Non-randomised experimental study | Community pharmacist | No | Evaluation – Counselling and assessment of patients with UTI symptoms and antimicrobial prescribing or changing of treatment when appropriate | - *Clinical cure:* achieved in 88.9% of patients (610/686 completing follow-up). - *Adverse events*: 7.2 % (54/750, mostly transient), 48/54 continued their medication. - *Patient adherence to therapy*: most patients (656/686; 96.5%) took their medication as prescribed. - *Number of follow-ups per patient:* 1.1 (SD 0.2) - *Treatment failures*: 1.4% (10/688): 6 due to complications, of which 2 were pyelonephritis. - *Time from decision to seek care until seen by pharmacists*: 1.7 (SD 2.4) days vs 2.8 (SD 3.8) days for a physician (p = 0.0153) - *Patient satisfaction*: very high levels of satisfaction for the care they received, as well as for trust and accessibility of the pharmacist. |
| Beahm et al.,  2021 ^53^ | Canada | Antimicrobial utilization and stewardship in patients with uncomplicated urinary tract infections managed by pharmacists in the community: A sub-study of the RxOUTMAP trial. | To evaluate the appropriateness of pharmacists' antibacterial prescribing for patients with uncomplicated UTI. | Cohort study | Community pharmacist | No | Evaluation – Patient with UTI symptoms assessment and counselling, including antimicrobial prescribing or changing when appropriate. | - *Prescriptions concordance with guidelines*: therapy was guideline concordant for 95.1% of patients (624/656) in the pharmacist-initiated arm and 35.1% of patients (33/94) in the physician-initiated arm (p < 0.001). For guideline-discordant therapy started by physicians, pharmacists prescribed to optimise therapy for 45.9% (28/61) of patients. |
| Beaucage et al.,  2006 ^51^ | Canada | Telephone follow-up of patients receiving antibiotic prescriptions from community pharmacies. | To evaluate the impact in the community setting of a pharmacist telephone follow-up intervention on the number of infectious symptoms and the severity of infection, as well as on the number and management of drug-related problems, adherence to treatment, patient satisfaction, and incremental direct costs of the intervention. | Randomised controlled trial | Community pharmacist | No | Evaluation – Educational intervention provided through telephone follow-up to patients presenting with and dispensed antibiotic prescriptions, compared with a control arm receiving usual care. | - *Proportion of patients with at least one detected drug-related problem*: intervention arm = 53% (67/126), control arm = 8% (10/129). - *Oral recommendations provided*: intervention arm = 52% (66/126), control arm = 6% (8/129). - *Recognised and study-specific pharmaceutical advice*: intervention arm = 10% (12/126) and 5% (6/126), control arm = 2% (3/126) and 1% (1/126). - *Mean difference in the change in the number of infectious symptoms and in the infection severity score between the two groups*: not statistically significant (-0.24 symptom, 95% confidence interval [CI] = -1.22 to 0.74) (-0.05 unit, 95% CI = -0.35 to 0.25) - *Adherence to treatment*: no difference between the two groups. - *Patient satisfaction*: no difference between the two groups. - *Cost of intervention*: incremental direct costs from $2.65 to $5.11 (Canadian dollars) per patient, depending on whether cognitive services were reimbursed. |
| Booth et al., 2013 ^43^ | United Kingdom, Scotland | Antibiotic treatment of urinary tract infection by community pharmacists: a cross-sectional study. | To compare the care pathway of patients with UTI symptoms attending GP services with those receiving management, including trimethoprim supply under PGD, via CPs. | Mixed methods study | Community pharmacist | No | Feasibility - Patient with UTI symptoms assessment and management, including possible antimicrobial initiation under PGD. | - *Adherence to guidance by GPs and pharmacists*: GP adherence to local guidelines: 56% (54/97 patients); pharmacists’ antibiotic prescriptions meeting PGD criteria: 76% (31/41). - *Patients’ satisfaction and demand for service, pharmacists’ desire to provide the service*: there was demand and support, from patients and pharmacists, for access to antibiotic treatments for UTIs, without prescription, through CPs. |
| Buchanan et al.,  2020 ^44^ | United Kingdom, England | The testing of people with any risk factor for hepatitis C in community pharmacies is cost-effective. | To report outcomes from a 24-month study of HCV targeted testing in CPs in a UK population and present a cost-effectiveness analysis that accounts for the additional costs of testing. | Non-randomised experimental study | Community pharmacist | No | Evaluation – Screening of high-risk patients for detection of potential HCV infections through pharmacist administered testing. | - *Virological response at 3 months*: 13/186 (7%) patients tested positive and 6 were treated, all of them achieved a sustained virological response at 3 months. - *Cost-effectiveness of HCV testing in CP*: overall cost of the testing and treatment intervention was £242183, and the ICER for the service was £3689 per QALY gained. If screening had been restricted to just people with a history of injecting drug use, the ICER would have been £4865 per QALY gained. |
| Byrd et al., 2019 ^30^ | United States | Retention in HIV Care Among Participants in the Patient-Centered HIV Care Model: A Collaboration Between Community-Based Pharmacists and Primary Medical Providers. | To develop and to implement a model of HIV care that integrates community-based pharmacists and clinic medical providers to provide patient-centred care for people with HIV. | Cohort study | Community pharmacist | No | Evaluation – Supporting HIV therapy management, including patient counselling, adherence monitoring and provision of drug-specific information. | - *Retention rate in HIV care pre- and post-implementation* (680 and 625 people included): 12.9% improvement post-implementation (60.7-68.5%, p = 0.002). The largest improvement was seen among non-Hispanic black persons, 22.6% increase (59.7-73.2%, p < 0.001). - *Adherence to ART and levels of HIV viral suppression*: not reported |
| Demorè et al.,  2018 ^56^ | France | Rapid antigen test use for the management of group A streptococcal pharyngitis in community pharmacies. | To test the feasibility, benefit and acceptance of a CP-based AMS intervention based on rapid antigen test use in adult patients with sore throat. | Non-randomised experimental study | Community pharmacist | No | Feasibility - Screening through POCT of patients presenting with sore throat, potentially caused by GAS infection. | - *Patients’ consultation rate after rapid antigen tests*: 8.3% (28/336) of tests were positive, 10/28 returned the follow-up form; 10/10 patients consulted a GP and were prescribed an antibiotic; 96.5% (110/114) of patients with negative results and not having any other reason to seek for GP's advice did not consult. - *Adherence to guidelines*: 100% of positive patients received an antibiotic and 96.5% of negative patients did not consult a GP and therefore did not receive an antibiotic. - *Patients’ acceptance of intervention*: all participants found the intervention useful. - *Time to complete the protocol*: pharmacists spent 6-15 min to perform the intervention. - *Pharmacists’ feedback*: 98.6% (73/74) of pharmacists giving feedback declared to be ready to implement this intervention in daily practice, if endorsed and reimbursed. |
| Göktay et al.,  2013 ^63^ | Turkey | The role of patient education in adherence to antibiotic therapy in primary care | To investigate whether patient education, as a clinical pharmacy related practice, given to patients prescribed antibiotics for any type of infections at the beginning of the treatment in a CP, is effective on adherence or not. | Non-randomised experimental study | Community pharmacist | No | Evaluation - Educational intervention with a group of patients receiving additional information regarding their antibiotic therapy and a control group where only information about the dosage regimen prescribed by the GP is provided. | - *Adherence to medication in terms of completion of treatment and appropriate timing*: 83.9% (26/31 patients) treatment completion in the study group vs 75.9% (22/29) in the control group; 80.6% (25/31) timing adherence in the study group vs 65.5% (19/29) in the control group (both p > 0.05). Patients who were prescribed a once daily dose regimen for a short duration were found more adherent in terms both of dose-taking (self-administration) and dose-timing (p < 0.05). |
| Havens et al.,  2019 ^31^ | United States | Acceptability and feasibility of a pharmacist-led HIV pre-exposure prophylaxis (PrEP) program in the Midwestern United States. | To investigate the acceptability and feasibility of a pharmacist-led HIV screening and PrEP programme (P-PrEP) for individuals at risk for HIV acquisition. | Non-randomised experimental study | Community pharmacist | No | Feasibility - Screening for HIV, including POCT for HIV and STIs, in individuals at risk of exposure and antimicrobial initiation when appropriate. | - *Number of patients initiated on PrEP*: 60 patients on 139 visits, (27/60 in CP). - *Retention in PrEP care*: participant retention at 3, 6, 9, and 12 months was 73% (44/60), 58% (35/60), 43% (26/60), and 28% (17/60), respectively. - *Adherence to treatment*: 93% mean medication possession ratio among retained participants. - *Patients’ satisfaction*: 100% of the participants who completed the patient satisfaction questionnaire would recommend the P-PrEP programme. - *Pharmacists’ satisfaction*: pharmacists reported feeling comfortable performing POCT and rarely reported feeling uncomfortable (3/139, 2.2%) during PrEP visits or experiencing workflow disruption (1/139, 0.7%). |
| Hayes et al., 2022 ^45^ | United Kingdom, England | Mixed-Method Evaluation of a Community Pharmacy Antimicrobial Stewardship Intervention (PAMSI). | To evaluate staff AMS behaviours before and after using PAMSI, including the checking of antibiotic appropriateness and providing patients with tailored infection and antibiotic use advice based on identified need. Secondary aims were to identify facilitators and barriers to intervention implementation. | Mixed methods study | Community pharmacist; other unspecified CP team members | Yes (COM-B model) | Evaluation - Educational intervention including providing patients with detailed antibiotic therapy information supported by information leaflets, checking for antibiotic appropriateness and querying with prescriber as necessary. | - *Reported checking of antibiotic appropriateness*: strong evidence of increased checking of appropriateness post-intervention (from 33/81 to 61/81, 41% to 79%, p < 0.001). - *Reported provision of patient information on antibiotic resistance, adherence, self-care, and safety-netting, as well as reported queries to antibiotic prescribers*: strong evidence of increase on antibiotic resistance (p < 0.001), importance of antibiotic adherence (p < 0.01) and self-care advice (p < 0,001). Staff reported feeling empowered querying an antibiotic prescription with a prescribing clinician post-intervention, but no statistical significance was found. |
| Heringa et al.,  2017 ^62^ | The Netherlands | Clinical Decision Support and Optional Point of Care Testing of Renal Function for Safe Use of Antibiotics in Elderly Patients: A Retrospective Study in Community Pharmacy Practice. | To investigate the management of drug therapy alerts on safe use of antibiotics in elderly patients with (potential) renal impairment and the contribution of optional POCT of renal function (creatinine) in daily CP practice. | Cross-sectional study | Community pharmacist | No | Evaluation - POCT for creatinine when required by software alerts and provision of advice for modification of antimicrobial treatment when appropriate. | - *Number of prescriptions where alerts were generated due to impaired renal function or missing data*: 88391 alerts for 64763 patients. In most of the cases, (82,5% of the alerts) pharmacists obtained or already had information on renal function. - *Number of patients requiring a POCT*: pharmacists performed 1988 POCTs (2.2% of the alerts). - *Pharmacist prescription modifications due to impaired renal function*: 15 out of 1988 POCTs (0.8%). |
| Hess et al., 2009 ^32^ | United States | Isoniazid completion rates for latent tuberculosis infection among college students managed by a community pharmacist. | To determine 6- and 9-month Isoniazid completion rates as well as the characteristics of treatment completers versus noncompleters in a CP latent tuberculosis infection clinic. | Cohort study | Community pharmacist | No | Feasibility - Patient counselling and medication adherence monitoring among college students with tuberculosis infection on Isoniazid. | - *6-month (defined as taking 180 tablets in the period) completion rate*: 67% (233/348 patients). - *9-month (270 tablets in a 9- to 12-month period) completion rate*: 59% (205/348). - *Reported ADEs' rate*: 21.6% (75/348), with only 7.9% referring to a student health physician for further evaluation. - *Characteristics of completers vs noncompleters*: noncompleters were more likely to have developed a rash (p = 0.03) or experienced fatigue (p = 0.04) than completers. - *Average time to treatment completion*: not reported. |
| Hirsch et al., 2009 ^33^ | United States | Evaluation of the first year of a pilot program in community pharmacy: HIV/AIDS medication therapy management for Medi-Cal beneficiaries. | To describe and compare patients filling prescriptions at pilot CPs (providing medication therapy management) vs other CPs with respect to: (a) patient characteristics; (b) intermediate outcomes including the number and type of ART medication regimens, rates of adherence, use of contraindicated ART regimens, and occurrence of opportunistic infections; and (c) pharmacy and medical costs. | Cohort study | Community pharmacist | No | Evaluation - HIV therapy management, including patient counselling, adherence monitoring and provision of drug-specific information. | - *ART adherence levels*: 56.3% in the pilot CPs (762/1353 patients) vs 38.1% (2157/5665) in other CPs (p < 0.001). (56.8% vs. 34.2%, p < 0.001). - *ART medication regimen strategy*: 63.8% (863/1353) of pilot CP patients were on protease inhibitor-based ART medication regimens vs 54.8% (3103/5665) in other CPs; 56.8% (768/1353) of pilot CP patients remained on a single type of ART therapy vs 34.2% (1938/5665) in other CPs; fewer pilot CP patients used contraindicated regimens (11.6% vs. 16.6%). For all data, p < 0.001. - *Decrease in occurrence of opportunistic infections*: 28.2% (381/1353) in pilot CPs vs. 26.1% (1478/5665) in other CPs (p = 0.121). - *Costs reduction through more rational medication usage and reduced need for medical services*: the total mean [standard error] annual health care cost per patient was 10% higher in pilot CPs than in other CPs ($40,596 [$889] vs. $36,937 [$479], p = 0.001). |
| Hirsch et al., 2011 ^34^ | United States | Antiretroviral therapy adherence, medication use, and health care costs during 3 years of a community pharmacy medication therapy management program for Medi-Cal beneficiaries with HIV/AIDS. | To examine the association between use of CPs participating in a medication therapy management pilot programme and adherence to ART regimens in a sample of patients with HIV/AIDS. | Cohort study | Community pharmacist | No | Evaluation - HIV therapy management, including patient counselling, adherence monitoring and provision of drug-specific information. | - *Adherence rate to ART*: A significantly higher percentage of patients at pilot CPs were adherent to their ART compared to nonpilot CP patients: in year 2007, 69.4% (436/628) vs. 47.3% (759/1606) (p < 0.001). - *Type of ART medication regimens*: pilot CP patients were more likely to stay on a single ART regimen: year 2007, 71.7% (450/628) vs. 49.1% (788/1606) (p < 0.001) - *Contraindicated ART regimens*: pilot CP patients were less likely to use them compared to other CPs: year 2007, 8.9% (56/628) vs. 12.2% (196/1606) (p = 0.027). - *Occurrence of opportunistic infections*: rates were similar between groups (≈ 35%). - *ART and non-ART medication costs*: no significant differences in total healthcare costs (2007: $38983 vs. $38856, p = 0.915). Non-ART medication costs were higher for pilot CP patients (30%-40% more), but inpatient service costs were lower (2007: $3083 vs. $5186, p < 0.001). |
| Ikwuobe et al.,  2013 ^65^ | Nigeria | The impact of rapid malaria diagnostic tests upon anti-malarial sales in community pharmacies in Gwagwalada, Nigeria. | To assess the incidence of malaria among unwell patients seeking anti-malarial treatment in two CPs in Nigeria and measure the impact rapid diagnostic tests have on anti-malarial sales. | Non-randomised experimental study | Community pharmacist; study nurse (conducting tests when requested to reduce pharmacists’ workload) | No | Evaluation – Screening through POCT for malaria of symptomatic patients. | - *Impact of rapid diagnostic tests on anti-malarial sales:* 58.1% (360/619) of patients tested in the intervention CPs purchased an antimalarial vs 100% (607/607) in the control CPs; 42% chance reduction (95% CI: 38%-46%). |
| Kawachi et al.,  2017 ^58^ | Japan | The detection of influenza virus at the community pharmacy to improve the management of local residents with influenza or influenza-like disease | To evaluate the effects of CP-based influenza virus screening and prevention measures. | Non-randomised experimental study | Community pharmacist | No | Evaluation - Screening for influenza of symptomatic patients through POCT and provision of recommendations appropriate to the test results. | - *Consistency between CP and clinic screening test results*: Influenza virus was found in 28.8% (15/52) of samples. Testing at the clinic confirmed the results of testing at the pharmacy. - *Patients’ satisfaction with the service*: 22 out of 24 (91,7%) participants responding to the mail-in survey indicated their satisfaction with the CP-based screening. |
| Klepser et al.,  2016 ^35^ | United States | Community pharmacist-physician collaborative streptococcal pharyngitis management program. | To describe a CP-based, collaborative physician-pharmacist GAS management programme through characterisation of the patient population and service patterns. | Cohort study | Community pharmacist | No | Feasibility – Screening through POCT for GAS of symptomatic patients and counselling based on the test result, including possible antimicrobial initiation under the Collaborative Practice Agreement. | - *Positive GAS rapid diagnostic tests ratio*: of 273 patients (86.4%) eligible for testing, 48 (17.6%) had positive test results. - *Number of antibiotic prescriptions*: 46/273 (16.8%) received amoxicillin or azithromycin per the Collaborative Practice Agreement. - *Recorded treatment decisions*: if the patient had a negative test the pharmacist discussed the findings with the patient and recommended appropriate OTC products. An encounter summary was generated and forwarded to the primary care provider. |
| Klepser et al.,  2019 ^36^ | United States | Evaluation of a community pharmacy-based influenza and group A streptococcal pharyngitis disease management program using polymerase chain reaction point-of-care testing. | To demonstrate the feasibility of implementing a Polymerase Chain Reaction (PCR) molecular test into a CP setting as part of a collaborative influenza and GAS disease management programme. | Non-randomised experimental study | Community pharmacist | No | Feasibility - Screening through POCT for GAS and influenza of symptomatic patients and counselling based on the test result, including possible antimicrobial initiation under the Collaborative Practice Agreement. | - *Number of patients tested*: 202 patients at the 2 CPs (116 for influenza, 46 for GAS, and 43 for both). - *POCT results*: 60 (38%) tested positive for influenza, and 16 (18%) for GAS. - *Consequent treatment recorded*: Oseltamivir was prescribed for 51/60 (85%) Influenza-positive patients; Amoxicillin (14/16) and Azithromycin (2/16) were prescribed for GAS-positive patients. No patient testing negative was dispensed an antibiotic. |
| Lambert et al.,  2005 ^67^ | Bolivia | Collaboration between private pharmacies and National Tuberculosis Programme: an intervention in Bolivia. | To evaluate the potential of a collaboration between the National Tuberculosis Programme and CPs in Bolivia. | Cross sectional study | Community pharmacist | No | Feasibility - Referral to the National Tuberculosis Programme of patients with chronic cough. | - *Number of referrals to National Tuberculosis Programme by CPs*: 26 of 70 CPs (38%) referred a total of 41 patients for screening (i.e. an average of 0.29 patients per CP and per month). - *Screening and diagnosis rates*: 11 of 41 patients referred to the programme (27%) were screened and 3 of 11 (27%) diagnosed with smear-positive tuberculosis. |
| Madaras-Kelly et al., 2006 ^37^ | United States | Experience with a clinical decision support system in community pharmacies to recommend narrow-spectrum antimicrobials, nonantimicrobial prescriptions, and OTC products to decrease broad-spectrum antimicrobial use. | To determine the feasibility of a protocol-driven CP intervention that was designed to decrease broad-spectrum antimicrobial use in patients with upper RTIs. | Cohort study | Community pharmacist | No | Feasibility – Assessment of patients with a prescription for a broad-spectrum antimicrobial and contact with primary care provider to suggest antimicrobial prescription modification to narrow-spectrum antimicrobials when appropriate. | - *Number of patients willing to discuss their symptoms with the pharmacist*: 192 subjects presented with prescriptions for broad spectrum antimicrobials and symptoms of RTIs. 3% of patients (6/192) declined to discuss their symptoms and treatment with the pharmacist. - *Number of patients accepting pharmacists’ contact with their primary care provider*: 7% (4/59 eligible subjects) of patients agreed to the intervention. - *Number of primary care providers altering therapies following contact with the pharmacist:* it was possible to contact the primary care provider in 3 of the 4 cases. Two of the 3 contacted providers accepted the recommendation. In both cases, a narrow-spectrum, inexpensive, antimicrobial was substituted. |
| Mantzourani et al.,  2022 ^46^ | United Kingdom, Wales | Characteristics of the sore throat test and treat service in community pharmacies (STREP) in Wales: cross-sectional analysis of 11304 consultations using anonymized electronic pharmacy records. | To describe key characteristics of the service, service users and antibiotic supply, to better  understand the value and need for the service, and its wider implications for primary care services. | Cross sectional study | Community pharmacist | No | Evaluation - Screening for GAS, using POCT, of symptomatic patients and patient counselling, including antimicrobial initiation and supply when appropriate. | - *Number of Sore Throat Test and Treat consultations and demographics*: 11304 in service users aged 6 years and over, with a median age of 25 years (IQR: 12 to 44). - *Proportion of service users reconsulting with a pharmacist for sore throat*: a rapid test was undertaken in 76.7% of consultations 8665/11304), 28.9% positive tests (2503/8665). - *Antibiotic supply rates*: 21.3% (2406/8665). - *Number and rates of different referral sources to the service*: 5580 (49.4%) service users were referred by their GP, 46.6% self-referred. - *Proportion of consultations using Centor/FeverPAIN scores*: pharmacists managed 10290 (91%) consultations (92.1% with FeverPAIN, 7.9% Centor score) in the CP. - *Number and proportion of CP referrals to GPs and emergency services*: 937 (9.3%) referrals to a GP and 27 (0.2%) to the Emergency Department. - *Percentage of service users who would have used alternative health services had service not been available*: 92.7% (10482/11304) of patients would have contacted their GP. |
| Merks et al., 2019 ^60^ | Poland | Patients' Perspective And Usefulness Of Pictograms In Short-Term Antibiotic Therapy - Multicenter, Randomized Trial. | To evaluate the practical utility of pharmaceutical pictograms in routine practice in CP. | Randomised controlled trial | Community pharmacist | No | Evaluation - Educational intervention with a study group in which patients were provided antibiotic accompanied by pictograms containing information about drug regiment and a control group in which patients received usual care. | - *Complete use of the whole package of medication*: in the control group, 16.7% (17/102) of participants discontinued therapy before completing the package compared with 13.4% (13/97) in the study group. - *Taking the recommended dose twice a day*: in the control group, 81.4% (83/102) of patients reported that they always took the medication twice a day (as recommended) compared with 80.4% (78/97) of patients in the study group. - *Patients’ perspectives on medical information obtained by the pharmacist*: the Net Promoter Score was higher for pharmacy practice with than without pictograms (71.3% vs 51.5%, respectively, p < 0.005). |
| Munoz et al.,  2014 ^61^ | Spain | The effect of an educational intervention to improve patient antibiotic adherence during dispensing in a community pharmacy. | To evaluate the benefit of an oral educational intervention,  in terms of increased treatment adherence and symptom  improvement or resolution, versus ‘‘routine pharmaceutical care’’. | Non-randomised experimental study | Community pharmacist | No | Evaluation - Educational intervention, with patients in the study group receiving detailed verbal information about their treatment and patients in the control group receiving usual care. | - *Medication adherence*: at the end of the study, treatment adherence in the control group was 48.4% (30/62, CI: 36.4-60.6), compared with 67.2% (43/64, CI: 55.0-77.4) in the intervention group (p = 0.033). - *Patients’ perceived health*: no significant difference, although it was higher in the intervention group, with reports of being “totally cured’’ in 54.7% (95% IC = 42.6-66.3) in the intervention group and in 46.8% (95% IC = 34.9-59.0) in the control group (p = 0.297) |
| Murphy et al.,  2012 ^38^ | United States | Impact of HIV-specialized pharmacies on adherence and persistence with antiretroviral therapy. | To assess adherence to ART, as well as persistence with ART for patients utilising HIV-specialised pharmacies compared to traditional CPs. | Non-randomised experimental study | Community pharmacist | No | Evaluation - HIV therapy management tailored to the individual patient, including medication review, adherence assessment and refill synchronisation. | - *Medication adherence*: specialised pharmacy (7064 patients in each group after propensity score) users had a significantly greater mean (74.1% vs 69.2%, p < 0.0001) and median (90.3% vs 86.3%, p < 0.0001) proportion of days covered. A greater percentage of patients in the specialised group were able to obtain a proportion of days covered of 95% or better (39.3% versus 35.5%). - *Medication persistence*: patients in the specialised group were significantly more persistent (p = 0.0117). |
| Northey et al.,  2015 ^54^ | Australia | Patients' antibiotic knowledge: a trial assessing the impact of verbal education. | To assess the effectiveness of involving CP staff in patient education about antibiotic resistance, thus improving antibiotic knowledge. | Randomised controlled trial | Community pharmacist | No | Feasibility - Educational intervention, with patients in the study group receiving detailed verbal information and patients in the control group receiving usual care. | - *Knowledge of patients about antibiotics and antibiotic resistance*: significant increase 1 month after receiving the intervention (antibiotic knowledge score: 33.3 ± 40.8) as compared with patients not receiving verbal antibiotic education (-5.1 ± 23.0) (34 patients, p = 0.008). |
| O'Neill et al.,  2022 ^47^ | United Kingdom, Northern Ireland | C-reactive protein point of care testing in community pharmacy: Observational study of a Northern Ireland pilot. | To pilot point-of-care CRP testing for suspected RTI within CP in Northern Ireland. | Non-randomised experimental study | Community pharmacist | No | Feasibility - Screening for CRP, using POCT, of patients with symptoms indicative of RTI and patient counselling based on the test result. | - *Consultation outcomes*: regardless of the CRP test result (< 20 mg/L = 237 patients, between 20 and 100 mg/L = 85 patients, > 100 mg/L = 4 patients), similar proportions of patients received verbal advice and a patient information leaflet. Patients with a CRP test result between 20 and 100 mg/L were more likely to receive an additional OTC medicine (40%, 34/85). Patients with CRP levels 20-100 mg/L (33%, 28/85) or > 100 mg/L (100%, 4/4) were more likely to be referred to GPs. - *Reasons for GP referral*: 13/38 (34%) patients were considered high risk, 12/38 (32%) had persistent symptoms and 10/38 (26%) were systemically unwell. - *Antimicrobial prescribing rates*: in a subgroup (n=30) from 1 practice with 22/30 (73%) patients with CRP test results < 20mg/L, 15/30 (50%) patients had contact with the GP in relation to their acute cough and 13/30 (43%) had an antibiotic prescribed within 5 days. |
| Onwunduba et al.,  2023 ^66^ | Nigeria | Impact of point-of-care C-reactive protein testing intervention on non-prescription dispensing of antibiotics for respiratory tract infections in private community pharmacies in Nigeria: a cluster randomized controlled trial. | To ascertain if access to CRP test kits – and staff training them on how to use them accordingly in the management of RTI – in CPs can reduce non-prescription antibiotic dispensation for RTIs. | Non-randomised experimental study | Community pharmacist; pharmacy assistant | No | Feasibility - Screening for CRP, using POCT, of patients with symptoms indicative of RTI and patient counselling based on the test result. | - *Non-prescription antibiotic dispensing rate for RTIs*: 15.66% decrease (209/300 [intervention] vs 256/300 [control]) in the adjusted analysis (OR = 0.279, 95% CI = 0.107-0.726; p = 0.009). - *Proportion of visits in which relevant tests were conducted*: 21.7% (65/300) rate in the intervention group vs 0/300 in the control group. Antibiotics were not dispensed in 28/64 (43.8%) of the visits with a CRP test. |
| Pham et al., 2013 ^39^ | United States | A randomized, controlled study of an educational intervention to improve recall of auxiliary medication labeling and adherence to antibiotics. | To evaluate whether medication counselling with emphasis on auxiliary labels improves recall of auxiliary label information and adherence to medication schedules. | Randomised controlled trial | Community pharmacist | No | Evaluation - Educational intervention with patients in the study group receiving detailed verbal information and patients in the control group receiving usual care. Auxiliary labels were provided to all participants. | - *Patients’ recall of medication instructions based on auxiliary label information provided*: the rate (overall 39 patients) was high irrespective of whether they were in counselling or non-counselling group. Among those with incorrect recall, 7 out of 9 subjects received no counselling (p = 0.11). - *Medication adherence*: no statistically significant difference between groups, based on patients’ report through tablet counting. |
| Radley et al.,  2017 ^48^ | United Kingdom, Scotland | DOT-C: A cluster randomised feasibility trial evaluating directly observed anti-HCV therapy in a population receiving opioid substitute therapy from community pharmacy. | To address questions about increasing testing and uptake of treatment, through a simplified community pharmacist-led care pathway for patients with genotype 1 HCV and to incorporate CPs into the work of a multidisciplinary Managed Care Network. | Cluster Randomised controlled trial | Community pharmacist | No | Feasibility - Patient counselling and screening, including blood spot testing, for HCV in population at risk. In the intervention group, pharmacists continued patient assessment and could initiate and supply antiviral therapy. | - *Proportion of opioid-substitution-therapy patients accepting the offer of testing*: conventional pathway: 58 dried blood spot testing from 244 patients (24%). Pharmacist-led pathway: 94 tests from 262 patients (36%). Participants in the pharmacist-led pathway were more likely to take the test. (p < 0.003). - *Proportion of patients undertaking assessment for treatment: conventional pathway*: conventional pathway: 4 patients from 15 with new reactive tests (27%) attended clinic for assessment. Pharmacist-led treatment pathway: 20 patients from 26 with new reactive tests (77%) attended for assessment. Participants in the pharmacist-led pathway were more likely to proceed through the assessment for treatment (p < 0.002). - *Patients completing treatment*: 1 participant in the conventional pathway and 3 through the pharmacist-led pathway. - *Service pathway costs*: costs associated with the CP setting are around 1/4 of the cost of treating a patient in a conventional setting (assuming the same cost of treatment). |
| Shrestha et al.,  2020 ^40^ | United States | Costs and Cost-Effectiveness of the Patient-Centered HIV Care Model: A Collaboration Between Community-Based Pharmacists and Primary Medical Providers. | To assess the costs and cost-effectiveness of the patient-centred HIV care model based on primary data of programmatic costs and intervention effectiveness (ie, rates of viral suppression) and on the published estimates of HIV transmission rates and lifetime treatment costs. | Non-randomised experimental study (economic evaluation) | Community pharmacist; pharmacy technician | No | Evaluation - HIV therapy management, including medication review, adherence assessment and refill synchronisation. | - *Total intervention cost*: overall, the patient-centered HIV care model annual intervention cost for the 3 project sites was $ 226741. - *Average cost per patient, per patient visit, and incrementally cost per patient virally suppressed*: $813, $48, and $5039, respectively. - *Averted HIV transmissions*: 2.75 - *Lifetime savings for HIV treatment*: 12.22 QALYs and nearly $1.28 million. - *Cost-effectiveness of the intervention*: the intervention was cost saving overall and at each project site. |
| Sim et al., 2021 ^55^ | Australia | Point-of-care C-reactive protein testing to support the management of respiratory tract infections in community pharmacy: A feasibility study. | To evaluate the feasibility, based on clinical and operational outcomes, of point-of-care CRP testing to support Western Australian community pharmacists' management of RTIs. | Non-randomised experimental study | Community pharmacist; pharmacy assistant; pharmacy intern | No | Feasibility - Screening for CRP, using POCT, of patients with symptoms indicative of RTI and patient counselling based on the test result. | - *Pharmacist management based on routine assessment and CRP levels*: CRP levels among the 131 participants recruited were: < 5 mg/L (bacterial infection unlikely; n = 60; 45.8%); 5-19 mg/L (bacterial infection possible if suggestive routine assessment; n = 52; 39.7%) and 20-100 mg/L (bacterial infection likely if suggestive routine assessment; n = 19; 14.5%). Pharmacists' management included OTC medicines (131, 100%), self-care advice (125, 95.4%) and immediate GP referral (15, 11.5%). - *Rate of recovery*: 65% (76/117) of participants had recovered by Day 5. - *Service uptake*: the service was provided in 21.2% of eligible RTI presentations, service uptake rate of 28.1%. - *Patients’ perceptions of whether they needed an antibiotic*: post-CRP testing, 50.9% (58/114) of participants had changed perceptions regarding their need for antibiotics. - *Consumer satisfaction*: high (100%), most participants (93.4%, 123/131) would utilise the service again. |
| Thornley et al.,  2016 ^49^ | United Kingdom, England | A feasibility service evaluation of screening and treatment of group A streptococcal pharyngitis in community pharmacies. | To test the feasibility and benefit of a service run from CPs incorporating rapid antigen detection testing for patients 12 years and over presenting with sore throat symptoms according to Centor criteria. | Non-randomised experimental study | Community pharmacist; other unspecified CP team members | No | Feasibility - Screening for GAS, using POCT, of symptomatic patients and patient counselling, including antimicrobial initiation through PGD when appropriate. | - *Reduction of unnecessary antibiotic use*: 149/367 (40.6%) patients were eligible for throat swab testing. Of these, only 36/149 (24.2%) were positive for GAS. Antibiotics were supplied to 9.8% (n=36/367) of all patients accessing the service. - *Patients who would have otherwise seen the GP if the service had not been available (savings to the NHS)*: 60/123 (48.8%) patients not showing signs of a bacterial infection (Centor score < 3) would have gone to their GP. |
| Thornley et al.,  2020 ^50^ | United Kingdom, England | Evaluation of a community pharmacy-led test-and-treat service for women with uncomplicated lower urinary tract infection in England. | To evaluate the effectiveness and uptake of a lower UTI test-and-treat service for women presenting with urinary symptoms within a CP in supporting self-care and appropriate use of antibiotics and reducing demand on other NHS resources. | Non-randomised experimental study | Community pharmacist | No | Feasibility - Patient with UTI symptoms assessment supported by the results of a urine dipstick test previously provided. Antimicrobial initiation through PGD when appropriate. | - *Women who purchased the service and were prescribed an antibiotic*: Of the 617 women eligible to participate, 496 (80.4%) purchased the test. Lower UTI was found to be likely in 372/496 (75.0%) women, most of whom (360/372, 96.8%) were supplied an antibiotic by use of a PGD. - *Women who would have sought a GP appointment if the service had not been available*: 214/301 (71.1%) women who completed the service and responded to the question would have visited their GP and 116/301 (38.5%) would have used self-care with or without going to see their GP (116/301). |
| Treibich et al.,  2017 ^57^ | France | The expected and unexpected benefits of dispensing the exact number of pills. | To assess the feasibility and the real impact of a change in the method of dispensing antibiotics in French CPs for 14 antibiotics. | Randomised controlled trial | Community pharmacist | No | Feasibility - Supply of the exact number of pills for antibiotic treatment completion vs supply of the pre-packed antibiotic boxes in the control group. | - *Acceptance from patients*: acceptance for per-unit dispensing was 80,6% (907 patients in the treated group and 278 in the control group). - *Number of pills saved in the per-unit delivery mode*: the initial packaging of the drugs did not match with the prescription in 60% of cases and per-unit dispensing reduced by 9.9% the number of pills supplied. An average of 23 pills were supplied in the control group against 20 in the per-unit dispensing (p = 0.02). - *Quality of information regarding the appropriate use of medications received at the CP*: no difference was found between groups. - *Medication adherence*: 782/856 (91.4%) patients in the treated group vs 84/128 (65.6%) in the control group (p < 0.01). |
| Tung et al., 2018 ^41^ | United States | Implementation of a community pharmacy-based pre-exposure prophylaxis service: a novel model for pre-exposure prophylaxis care. | To describe the service and report initial experiences. | Non-randomised experimental study | Community pharmacist | No | Development - Screening for HIV and STIs through blood sampling in individuals at risk and tailored patient counselling, including advice on medication adherence, drug usage and HIV and STIs risk reduction. Antimicrobial initiation through collaborative agreement when appropriate. | - *Number of patients evaluated in the clinic*: 714 patients - *Number of patients started on PrEP treatment*: 695 (97.3%) patients; 513 (74%) began medication the same day as their initial appointment. - *Number of patients retained*: 90% of patients had a mean proportion of days covered greater than 80%, 19% of patients were lost to follow up, with an effective drop-out rate of 25%. - *Number of HIV seroconversions*: none in the service. |
| West and Cordina, 2019 ^59^ | Malta | Educational intervention to enhance adherence to short-term use of antibiotics. | To assess whether an intervention by community pharmacists supported by an educational leaflet enhances adherence and reduces costs in relation to wastage of unused antibiotics amongst patients taking short-term antibiotics in community and to determine a possible association between adherence and patients' general medicines' beliefs about medicines. | Randomised controlled trial followed by cross-sectional survey of patients who participated | Community pharmacist | Yes (NPT) | Evaluation - Educational intervention, with patients in the study group receiving detailed information about their treatment and patients in the control group receiving usual care. | - *Medication adherence*: 20/200 (20%) patients from intervention group and 48/200 (24%) from control were non-adherent (p = < 0.0005). - *Antibiotic wastage*: 2.8-fold more in the percentage cost of wasted antibiotics in control group. - *Association between adherence to antibiotics and patients’ general beliefs*: "general-benefit" belief was significantly higher for intervention group (p = 0.044). For control group, higher "general-overuse" beliefs were significantly associated with non-adherence (p = < 0.0005). |

CP = Community Pharmacy; RTI = Respiratory Tract Infection; GP = General Practitioner; COM-B: Capability, Opportunity, Motivation – Behaviour; ART = AntiRetroviral Therapy; UTI = Urinary Tract Infection; PGD =Patient Group Direction; HCV = Hepatitis-C Virus; QALY = Quality-Adjusted Life Years; ICER = Incremental Cost-Effectiveness Ratio; HIV = Human Immunodeficiency Virus; AMS = AntiMicrobial Stewardship; PrEP = Pre-Exposure Prophylaxis; POCT = Point-Of-Care Testing; ADE = Adverse Drug Event; GAS = Group A Streptococcus; CRP = C-Reactive Protein; NPT = Normalisation Process Theory
